# Cadmium inhibitory action leads to changes in structure of ferredoxin:NADP^+^ oxidoreductase

**DOI:** 10.1007/s10867-012-9262-z

**Published:** 2012-02-02

**Authors:** Joanna Grzyb, Mariusz Gagoś, Beata Myśliwa-Kurdziel, Monika Bojko, Wiesław I. Gruszecki, Andrzej Waloszek, Kazimierz Strzałka

**Affiliations:** 1 Department of Plant Physiology and Biochemistry, Faculty of Biochemistry, Biophysics and Biotechnology, Jagiellonian University, ul. Gronostajowa 7, 30-387 Krakow, Poland; 2 Laboratory of Biological Physics, Institute of Physics, PAS, al. Lotników 32/46, 02-668 Warsaw, Poland; 3 Department of Biophysics, University of Life Sciences in Lublin, Akademicka 13, 20-950 Lublin, Poland; 4 Department of Cell Biology, Institute of Biology, Maria Curie-Skłodowska University, 20-033 Lublin, Poland; 5 Department of Biophysics, Maria Sklodowska-Curie University, Lublin, Poland

**Keywords:** Ferredoxin:NADP^+^ oxidoreductase, Cadmium, Heavy metals, Secondary structure, Tertiary structure

## Abstract

This study deals with the influence of cadmium on the structure and function of ferredoxin:NADP^+^ oxidoreductase (FNR), one of the key photosynthetic enzymes. We describe changes in the secondary and tertiary structure of the enzyme upon the action of metal ions using circular dichroism measurements, Fourier transform infrared spectroscopy and fluorometry, both steady-state and time resolved. The decrease in FNR activity corresponds to a gentle unfolding of the protein, caused mostly by a nonspecific binding of metal ions to multiple sites all over the enzyme molecule. The final inhibition event is most probably related to a bond created between cadmium and cysteine in close proximity to the FNR active center. As a result, the flavin cofactor is released. The cadmium effect is compared to changes related to ionic strength and other ions known to interact with cysteine. The complete molecular mechanism of FNR inhibition by heavy metals is discussed.

**Electronic supplementary material** The online version of this article (doi:10.1007/s10867-012-9262-z) contains supplementary material, which is available to authorized users.

## Introduction

This paper is the second part of a study that addresses the question of the response of ferredoxin:NADP^+^ oxidoreductase (FNR) to increased concentrations of heavy metals, mainly cadmium. FNR is one of the key photosynthetic enzymes (for review, see e.g., [1, 2]). Isolated FNR is a two-domain monomer of molecular mass around 36 kDa. One domain is an NAD(P)-binding site with a typical dehydrogenase motif beta-alpha-beta. A second, N-terminal domain, rich in beta structure, noncovalently binds FAD, being a cofactor of the enzyme [3]. The active center of FNR consists mainly of Ser96, Glu312, and Tyr314. Tyrosine stabilizes FAD [3], while serine orients its isoalloxasine ring, enabling proton transfer to glutamate and other residues [4, 5].

In previous work [6], we proposed a mechanism for FNR–cadmium interaction deduced from enzymological investigation. There, we provided evidence for the existence of many (up to a few dozen) cadmium-binding sites of low affinity, which have a small impact on enzymatic activity, and probably one strongly binding site, which might be responsible for strong inhibitory effects. The explanation for the observed effects might be two-step inhibition: first, cadmium ion-dependent induction of some structural changes, which, subsequently, allow the binding of one of the Cd ions to cysteine(s) localized in a hidden cavity, most probably close to the reactive center of the enzyme. Here we focus on those structural changes in the protein molecule that occur due to the binding of a metal ion. Various tools common in structural biology make it possible to study both secondary and tertiary structure changes induced in a protein molecule after the action of a metal ion. Secondary structure is easily measured by circular dichroism (CD). This technique has already been applied to study cadmium-protein interactions for S-100b protein from a bovine brain [7] and yeast hexokinase [8]. However, the CD spectrum is measured in the ultraviolet to determine the relative content of secondary structure elements, and it may be strongly perturbed by any molecules absorbing in this region, including widely used buffers. For this reason, we applied Fourier-transform infrared spectroscopy (FTIR), a complementary technique [9] that has already been successfully applied to FNR investigations [10, 11]. Changes in the protein structure could also include alterations in tertiary structure, e.g., in the relative positioning of domains or helices, without any changes in stability of helices or *β*-sheets. This might result in an increase or decrease in the exposure of selected amino acid moieties. In the case of tryptophan and other aromatic residues, such changes in environment could be tracked by fluorescence, both steady-state and time-resolved [12]. Apart from tryptophan residues, the fluorescence of FAD, a natural fluorophore and the FNR cofactor, can be measured. An analysis of FAD fluorescence spectra might show the modification of its binding mode, including binding of substrate [13], protonation [14] and complete release from apoprotein [15, 16], and therefore it may support the hypothesis about the cadmium-binding site.

The aim of this work was to obtain data that would enable us to determine changes in FNR structure, which, together with previous enzymatic studies [6], would give a complete picture of FNR–Cd interaction and a molecular basis for further in vivo investigations.

## Materials

### Isolation and purification of FNR

Ferredoxin:NADP^+^ oxidoreductase and ferredoxin were isolated and purified from spinach leaves, obtained at a local market, in a procedure described in [17]. The concentration of protein was determined by the Bradford assay. The molarity of FNR was calculated using Mw = 36 kDa. The activity of isolated FNR was determined by an in vitro assay, as described in [17], with minor modifications. Reaction mixture for measuring diaphorase activity of FNR (in total volume 2 ml) contained 100 μM NADPH (Sigma-Aldrich, Schnelldorf, Germany), 25 μM DBMIB (Sigma-Aldrich, Schnelldorf, Germany) and 0.01 μM FNR, in a 40 mM Tris/HCl buffer, pH 8.7. The reaction rate was monitored with a dual wavelength spectrophotometer DW2000 SLM Aminco^®^ (USA) in kinetic mode (*λ* = 340 nm vs. *λ* = 700 nm) and the amount of oxidized NADPH was calculated from the extinction coefficient *ε*_340_ = 6.2 mM^ − 1^ cm^ − 1^.

## Fluorescence measurement

### Spectrofluorometry (steady-state)

Steady-state fluorescence of tryptophan residues and the FAD of FNR was recorded on a spectrofluorometer FluoroMax-P (Horiba Jobin Yvon Inc., Edison, NJ, USA), for 1.1 μM FNR in 40 mM Tris/HCl, pH 8.7 or 7.0. CdCl_2_ or NaCl were added from water stock solutions (50 mM for cadmium and copper salts, 100 mM for sodium salt). Final concentrations were 1.25 mM for cadmium, and 5 mM for sodium. The fluorescence emission spectra of FAD residues were recorded in a range of 480–650 nm, for excitation at 350 nm, and with excitation and emission slits of 3 nm; resolution and integration time were 1 nm and 0.2 s, respectively. The emission spectra of the tryptophan moiety were recorded in a range of 300–505 nm, for excitation at 280 nm. The excitation and emission slits were 2 nm. The resolution and integration time were 1 nm and 0.25 s, respectively.

The fluorescence excitation spectra of the FAD moiety were recorded between 290 and 500 nm, for emission at 520 nm. The excitation and emission slits were the same as in the case of the emission spectra, while the resolution and integration time were set at 0.5 nm and 0.1 s, respectively. The release of FAD from the protein was checked after 60 min of incubation for all experiments. For this purpose, the incubation mixtures were concentrated using centrifuge concentrators (Vivaspin, Littleton, MA, USA) with a cut-off limit of 10 kDa. The filtrate was examined using the parameters for the FAD fluorescence excitation spectra. Kinetics of tryptophan and FAD fluorescence emission were recorded successively, with no intervening delay. Tryptophan fluorescence was excited at *λ* = 280 nm and observed at *λ* = 340 nm whereas for FAD excitation and emission were *λ* = 450 nm and 520 nm, respectively. The excitation and emission slits were 2 nm, and fluorescence was recorded every 10 s with an integration time 0.5 s.

### Fluorescence lifetime

Fluorescence lifetime measurements were performed using a multifrequency, cross-correlation, phase and modulation fluorometer (model K2, ISS Instruments, Urbana, IL, USA) as described previously [18] with the suspension of glycogen in water as a *τ* = 0 reference.

The source of continuous light for excitation of fluorescence was a xenon lamp (300 W). The intensity of the excitation beam was modulated with a Pockels cell within a range of 2–200 MHz. Each measurement was performed for 12 frequencies. Trp and FAD fluorescence were excited at 280 and 450 nm, respectively, using a monochromator. The fluorescence of both fluorophores was observed through long-pass filters with a cut-off at 305 and 550 nm, respectively. The maximal errors in the measured phase shift and modulation were ±0.3 and ±0.006, respectively. Phase and modulation data were analyzed with ISS software (ISS, Greenville, USA) and a non-linear least-squares method assuming a discrete decay in fluorescence. The FNR concentration used for measurements was 2.2 μM in 40 mM Tris/HCl, pH 8.7 or 7.5. Cadmium chloride was applied from a water stock solution (50 mM) to a final concentration of 1.6 mM. The FNR:Cd ratio in the sample was adjusted so as to observe 50% inhibition of diaphorase activity [6], after 30 min incubation in 20°C.

### Lipid bilayers

A bilayer of lipids was formed between the water surface (buffered with 25 mM HEPES/NaOH pH 8.7) and the surface of a germanium (Ge) crystal by the “attach” technique [19, 20]. The equipment used made possible a controlled shifting of Ge. A dish was filled with 12.3 ml of buffer, and a volume of 25 μl of 5 mM DGDG in a chloroform stock solution was deposited to obtain *π* = 20 mN/m (previously tested using a tensiometer). The Ge crystal was shifted to touch the monolayer, and picked up. A second volume of 10 μl of DGDG stock solution was added to obtain again *π* = 20 mN/m and the Ge crystal was again moved down to form a bilayer. FNR was injected into the water phase, beneath the bilayer formed on the Ge crystal, with a microsyringe. The FTIR spectra of the bilayer were recorded after injection and during subsequent 10-min periods, in a range of 400–4,000 cm^ − 1^, with a resolution of 4 cm^ − 1^. Typically 36 scans were accumulated, Fourier transformed, and averaged. As a background, the spectrum just after injection of FNR was recorded (time zero). Cadmium chloride was injected as a 50-mM water stock solution, after a time of 90 min (to obtain a stable Amide I band of FNR).

### FTIR spectra of FNR partially hydrated film

0.2 μM FNR in 40 mM Tris/HCl pH 8.7 was incubated with 1.25 mM CdCl_2_ for 30 min at room temperature, and dialyzed overnight against 40 mM HEPES/NaOH, pH 7.5. For the control treatment, the protein was incubated and dialyzed as described, but water was added instead of cadmium salt solution. This protein solution was concentrated on a ZnSe crystal with a gentle stream of nitrogen. Final drying was done in a stream of argon, filling the FTIR spectrometer (Vektor 33, Bruker GmbH, Karlsruhe, Germany). FTIR spectra were recorded in a range of 400–4000 cm^ − 1^, with a resolution of 1 cm^ − 1^, and 36 scans were accumulated, averaged and Fourier transformed. As the background, a spectrum of clean crystal was used.

### Circular dichroism

CD spectra were measured with a Jasco 810 Spectropolarimeter (Jasco, Research Ltd, Easton, MD, USA) in a range of 175–260 nm, scan speed 10 nm/min, 5 scan accumulation. The FNR stock solution was dialyzed against a 25 mM Hepes buffer, pH 7.0. An aliquot (25 μl) of dialyzed stock solution was mixed with 275 μl of the respective buffer (25 mM Hepes/NaOH, pH 7.0 or 8.7). For the CD spectra of FNR in the presence of metal ions, respective volumes of CdCl_2_ were added from 50 mM (or 200 mM for NaCl) stock solutions or an equivalent volume of water for control. For the CD spectra of the protein in the presence of liposomes, the SUV in respective buffers were added. Spectra were analyzed by free software CDPro [21].

### Other chemicals

The water used for spectrophotometric and monolayer experiments was MiliQ quality. MGDG and DGDG came from Lipid Products (Redhill, UK). HEPES was from Sigma (Germany). Other chemicals were purchased from Polskie Odczynniki Chemiczne, Poland, and were of analytical grade.

## Results and discussion

In the paper describing enzymatic study of Cd inhibition [6], we found that cadmium binds at multiple sites on the FNR molecule, although only one atom is most probably really tightly bound and resistant to dialysis, which is enough to cause inhibition. However, the presence of other cadmium ions was necessary during the incubation time. In this part of the study, we want to prove that they induce the conformational changes necessary for binding this particular Cd^2 + ^ ion in the active center of the enzyme.

### Changes in secondary structure of FNR induced by cadmium

The most simple tool for studying structural changes in a protein is circular dichroism in the UV region. This does impose certain restrictions, among others the use of a phosphate buffer for obtaining good quality spectra. However, in the presence of cadmium, this was not possible. To obtain information on the influence of cadmium on the changes in the secondary structure of FNR, we used a HEPES buffer. As found in the previous paper [6], at pH 8.7 cadmium ions strongly inhibited FNR activity, but at pH 7.0 there was almost no inhibition noted. For that reason, we compared measurements performed at both pH values to discriminate between events related just to nonspecific interaction with charged ions and the event directly responsible for loss of FNR activity. Additionally, we also tested the structure in the presence of NaCl, to have a control for an ionic strength comparable to that of CdCl_2_.

The obtained spectra do not have a reliable shape in the *λ* < 200 nm, due to the presence of salts. The changes observed for pH 8.7 suggest a partial unfolding (an increase in the ratio of unordered to structured). It must be said, however, that a fit with any published algorithm (e.g., CDPro or JFit) involves a large fitting error and so does not help with the problem of alterations in secondary structure. For the details of the CD study and example spectra, see the Supplementary Material.

**Fig. 1:**
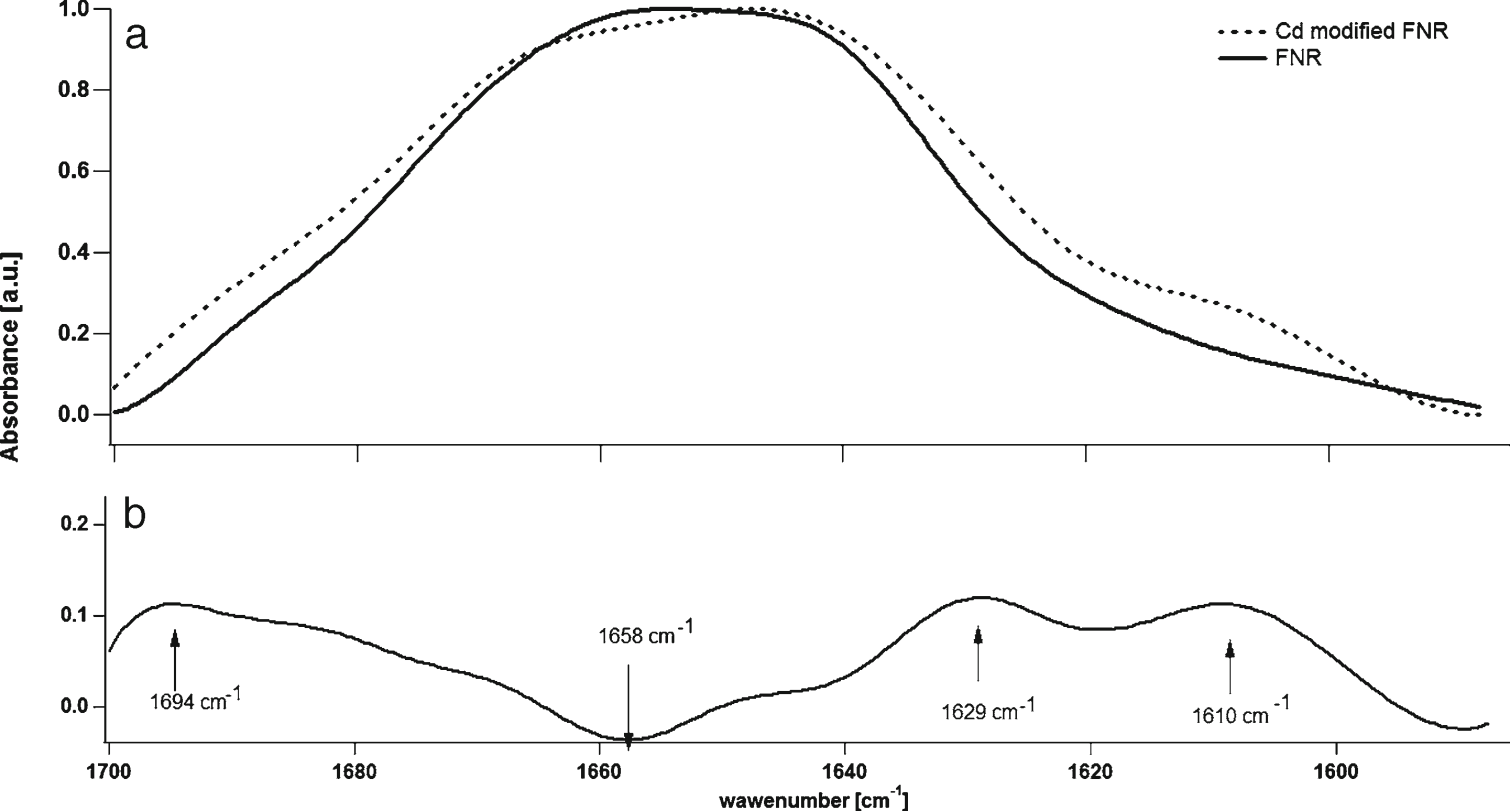
Changes in FNR FTIR spectrum (in the Amide I region), after cadmium treatment. FNR was incubated with cadmium, dialyzed, and deposited on Ge crystal (details described in Section 2). **a** Original spectra of the control and Cd-treated FNR, **b** difference spectrum (Cd-treated FNR − control sample)

More reliable results came from an examination of FNR secondary structure by FTIR. The spectra were compared for two variants:control FNR and the enzyme treated with cadmium. FNR treated with cadmium for 30 min was dialyzed overnight and deposited on a ZnSe crystal, as described above section. We have focused particularly on the Amide I region (1,600–1,700 cm^ − 1^) because it consists of well-defined bands, representing different types of protein secondary structure. Figure 1a presents the infrared absorption spectra of the control and cadmium-treated FNR, in the Amide I region. The most pronounced features in the difference spectrum (Fig. 1b) are the positive bands centered around 1,610 cm^ − 1^, 1,625 cm^ − 1^ and at 1,694 cm^ − 1^ and a slight decrease in the intensity of the band centered at 1,658 cm^ − 1^. Deconvolution of the Amide I allowed us to distinguish six components. The results of the detailed analysis are presented in Table 1. The structure of FNR shown here for a hydrated film of protein is compatible with the structure determined previously [10, 11]. However, some higher amount of coil was noticed, which could be a result of the FNR preparation procedure (prolonged incubation at room temperature and dialysis) corresponding also to a loss of enzymatic activity (about 20%) even in the control without cadmium ion treatment. The increased intensity of the band that appears in the high wavenumber wing of the Amide I band (at 1,680 cm^ − 1^ and higher) corresponding formally to an anti-parallel *β*-sheet structure, may also reflect FNR aggregation after Cd^2 + ^ treatment.

**Table 1:** Summary of results of Amide I band deconvolution for FNR treated and untreated with cadmium. Secondary structure type based on [22, 23]

FNR as partially hydrated film deposited on ZnSe crystal				FNR built in DGDG bilayer			
Wavenumber [cm^ − 1^]	Secondary structure type	Relative intensity		Wavenumber [cm^ − 1^]	Secondary structure type control	Relative intensity	
		Control	Cadmium-treated FNR			Control	Cadmium-treated FNR
1687	*β*-sheet	3.8	4.5	1673	*β*-sheet	0.5	1.13
1673	*β*-turn	18.3	18	1665	*β*-turn	13.9	11.4
1658	Coil	34	32	1649	*α*-helix	21.3	17.12
1641	*α*-helix	27.1	28	1634	*β*-sheet	36.4	41.1
1625	*β*-sheet/aggregate	12.2	10	1618	*β*-sheet/aggregate	20.7	20.1
1605	Backbone	4.1	7	1602	Backbone	7.12	9.1

**Fig. 2:**
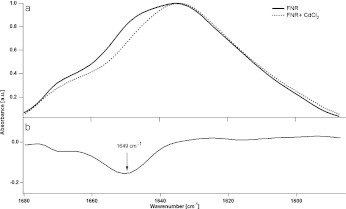
Changes in FTIR spectrum (in the Amide I region) of FNR, bound into the DGDG bilayer, after cadmium injection beneath the bilayer. **a** Original spectra of control (90 min after injection of FNR to water phase, buffered with 25 mM Hepes/NaOH, pH 8.7) and after injection of cadmium to this system (60 min after CdCl_2_ injection). **b** Difference spectrum (Cd-treated FNR − control sample)

However, the total structural changes are not high, and while such modified FNR is likely to contain just one bound cadmium [6] it might mean that disturbance in the protein secondary structure is not the main reason for the decrease in activity. Additional information about changes in the secondary structure of FNR upon cadmium treatment was obtained when the FTIR spectra was recorded for protein incorporated into the lipid (DGDG) bilayer. It is already known that FNR can attach and partially incorporate into such a bilayer [11], which may mimic its situation in vivo, when FNR is in contact with the thylakoid membrane. First, we checked that the presence of cadmium significantly influences neither the rate of FNR attachment to the membrane nor its detachment from the membrane (see description of monolayer experiment in Supplementary Material). After injection of the FNR beneath the bilayer, the Amide I band appeared, and maximal absorbance was reached after 60 min, corresponding to the plateau in increase in the monolayer experiment (see Supplementary Material). CdCl_2_ solution was then injected into the water phase, and the FTIR spectra were recorded at times of 0, 30, 60, and 90 min. The Amide I band was changed during FNR exposure to cadmium (Fig. 2a). The main change, as is shown in the difference spectrum (Fig. 2b), was a decrease in intensity at 1649 cm^ − 1^, compensated by an increase at 1634 cm^ − 1^. Details of deconvolution results are presented in Table 1. The observed changes, again, are not very high, confirming that conformational changes are not the main motor of cadmium-related inhibition of FNR.

### Tertiary structure changes as deduced from tryptophan fluorescence alteration

**Fig. 3:**
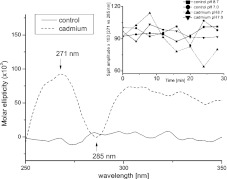
Difference spectra (starting point minus spectrum after 30 min), representing changes in the region between 250–350 nm of circular dichroism spectra of FNR (5.5 μM) treated with 4 mM CdCl_2_ (25 mM Hepes/NaOH, both pH 7.0 and 8.7) or in control (same conditions without cadmium added). *Inset* traces representing time dependence of split amplitude in control and cadmium-treated samples, both pH 8.7 and 7.0

While the changes in secondary structure might not be great, it is still possible that the relative position of secondary structure elements will be changed during the binding of cadmium. For example, the exposition of more hydrophobic regions after cadmium treatment has already been shown for the S-100b protein [7]. To check this possibility, we tracked the tertiary structure related part of FNR circular dichroism spectra, i.e., the 250–350 nm region. We observed a characteristic split with center about 280 nm, similar to described by [24]. Cadmium treatment at pH 8.7 decreased the intensity of the split, as a function of incubation time (Fig. 3). The corresponding change was not found in control (both pH 7.0 or 8.7) or during incubation with cadmium at pH 7.0.

The important information about changes in tertiary structure came from the study of tryptophan moiety fluorescence. There are six tryptophan residues in the FNR molecule, located in different parts of the protein—three moieties partially water accessible, and three other forming a cluster in the hydrophobic interior of the C-terminal domain. We assumed that relative changes in FNR structural elements may result in an alteration in tryptophan exposure to water, which might be very easily detected as a shift in the steady-state fluorescence spectrum [12]. Additionally, the conclusion about the fluorophore microenvironment may be strengthened by a time-resolved fluorescence study, showing changes in fluorescence decay.

**Fig. 4:**
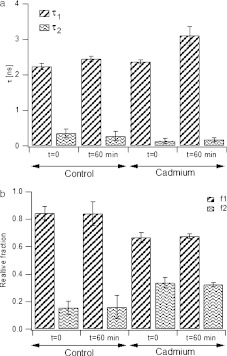
Comparison of FNR tryptophan fluorescence lifetimes (**a**) and their relative contribution to total fluorescence (**b**), in control FNR (2 μM) incubation and in the presence of CdCl_2_ (5 mM), in a 40 mM Tris/HCl pH 8.7. *Bars* show the average of three independent measurements and calculated standard deviation for time 0 and 60 min

Cadmium treatment caused a blue shift (3 nm) of the maximum of tryptophan fluorescence of the FNR molecule, but only at pH 8.7, when the inhibition is strong. Cadmium treatment also caused a decrease in the fluorescence intensity (compare Supplementary Fig. S4). At pH 7.0, there is no inhibition observed, and no changes in the spectra were detected. The observed effect is specific to cadmium (as it cannot be induced simply by increased ionic strength). The shift suggests that, unlike the case of a cadmium-treated S-100b protein [7], the fluorophore of FNR was moved to a more hydrophobic environment [25], or became surrounded by a water dipole oriented in a different way than for free tryptophan in a water solution [26]. The increase in the hydrophobicity of tryptophan moieties can also be confirmed by changes in tryptophan fluorescence decay. For the control (both at pH 8.7 and 7.0), two lifetimes could be determined—the first *τ*_1_ = 0.24 ± 0.09 ns, and the second, *τ*_2_  = 0.36 ± 0.12 ns (Fig. 4). The long component dominated, giving 85% of the relative fluorescence level (Fig. 4). In the time course of control incubation (FNR without cadmium added), the value and the relative content of both components changed slowly (not shown). The addition of cadmium, however, caused a change in this pattern—during the first 30 min of incubation *τ*_1_ increased to 3.2 ns, while *τ*_2_ did not change significantly. The relative content of the components was also altered—the long component still dominated, being 67% of total fluorescence, while the relative content of *τ*_2_ increased from 15% to 33% (Fig. 4).

The lifetime values found in our study are shorter than those for free tryptophan in a water solution (for detailed description, see [12]), which indicates a partial burying of the fluorophore (tryptophan residues) in the hydrophobic interior of the protein. After the cadmium treatment *τ*_1_ increased to 3 ns, with a relative fluorescence yield of 60%. Interestingly, the increase after cadmium treatment is closer to free tryptophan. The same increase, obtained for control incubation, but after significantly longer incubation at room temperature, suggests that tryptophan fluorescence decay depends on the destabilization of tertiary protein structure. Thus, cadmium binding to FNR residues induced a fast change in the tertiary structure. The study assigning lifetimes to specific Trp moieties in the FNR molecule is lacking; however, it is probable that the half-buried Trp residues (namely, Trp59, Trp182, and Trp199) have longer lifetimes (*τ*_1_), and the three tryptophan moieties forming a buried cluster (namely Trp259, Trp296, and Trp309) have shorter lifetime (*τ*_2_). The increase in length of *τ*_1_ might be then related to water-exposure of the partially exposed residues. Since *τ*_2_ did not change significantly, it might be postulated that cadmium cannot penetrate to the hydrophobic interior of the C-terminal domain, and the N-terminal domain structure is more influenced by cadmium action. The C-terminal domain has been already shown to be more stable, and folded even without the presence of FAD [16].

### Cadmium causes a release of the flavin cofactor of the FNR molecule

**Fig. 5:**
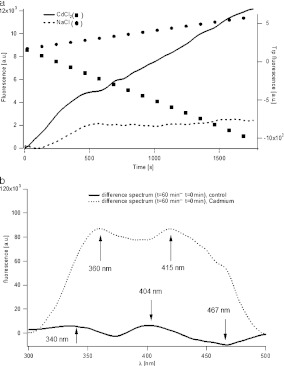
Changes in the fluorescence of the FAD cofactor and Trp residues of FNR (1 μM) after cadmium treatment: **a** kinetics of FAD fluorescence emission measured for *λ*_ex_ = 450 nm and *λ*_em_ = 520 nm, for the sample in the presence of one of the following salts: CdCl_2_ (1.25 mM) or NaCl (5 mM), in 40 mM Tris/HCl, pH 8.7 (corrected for small changes in control), with respective traces of Trp fluorescence emission measured for *λ*_ex_ = 280 nm and *λ*_em_ = 340 nm; **b** difference in FAD fluorescence excitation spectra (*λ*_em_ = 520 nm) measured at the beginning (0 min) and at the end (60 min) of incubation with CdCl_2_ (1.25 mM) or without CdCl_2_ (control)

The relative shift (opening) of FNR domains may result in the release of a cofactor, FAD, which is non-covalently bound, mainly to the C-terminal domain. Indeed, we detected that during incubation of FNR with cadmium, the intensity of the fluorescence of FAD significantly increased (Fig. 5a), which suggests a less tight binding or a release [15]. The increase in FAD fluorescence was correlated with a decrease in emission intensity of tryptophan moieties. Additionally, the positions of FAD excitation spectrum maxima were shifted during incubation at pH 8.7, indicating the appearance of a form with a fluorescence excitation maximum at 340 nm and 404 nm (Fig. 5b). The addition of cadmium to FNR at pH 7.0 did not change the shape of the FAD fluorescence excitation spectrum, although an increase in intensity was observed (not shown). Also, insignificant changes were found after the addition of NaCl, both at pH 8.7 and 7.0. The amount of free FAD was checked after a 60 min incubation. The incubation mixture was fractionated using concentrators with a membrane cut-off at 10 kDa. The filtrate gave a spectrum typical of free FAD (with maxima at 360 and 450 nm, not shown). In samples incubated with CdCl_2_ at pH 8.7 the amounts of free FAD were 4.5 times higher than after control incubation. For pH 7.0 or for incubation with NaCl at both pH values, no significant differences were detected. The changes in FAD fluorescence, both excitation and emission spectra, can be explained firstly by a destabilization of FAD binding (but not yet a release), and then a complete unbinding of the cofactor and the creation of FNR apoprotein [16]. In our case, this phenomenon may also be caused also by substitution of a cadmium ion for one FAD coordinated water molecule (compare crystal structure, [3]). The water molecules present in the active center of FNR may also be important for the catalytic mechanism of the enzyme [27].

The lack of drastic changes in either the cadmium-treated sample or the control at pH 7.0 indicates that the destabilization and release of FAD is directly connected to cadmium binding to cysteine(s), not to water substitution.

Existence of the Cd-S-cysteine bond could be confirmed by an analysis of the circular dichroism spectra in the 250–260 nm region (Fig. 3). The band at 253 nm is direct evidence of the existence of Cd-S chromophore, as observed e.g., for metallothioneins [28]. In the case of cadmium-treated FNR, we found an increase in that region, when compared to control. However, since the tertiary structure of FNR changed, strongly influencing the 250–350 nm spectral range, the confirmation of the Cd-S bond awaits further evidence.

## Conclusions

The present research, which includes a biophysical study of cadmium-induced changes in the secondary and tertiary structure of FNR, is complementary to the study which describes an alteration in FNR enzymatic function upon the action of cadmium [6].

In this paper, we show that cadmium induces conformational changes in the studied protein. Small alterations were noted in the secondary structure of FNR, but also rearrangements in tertiary structure were observed by tracking of tryptophan fluorescence. Not only cadmium but also other charged ions may cause similar effects, but only cadmium causes strong inhibition. This leads to the conclusion that the first inhibition event is related to “opening” of the protein molecule, which allows binding of the cadmium ion, which is responsible for the final inhibition event. This cadmium ion may probably be bound to cysteine residues close to the reactive center, which correlates with observed release of FAD cofactor during inhibition.

## Electronic Supplementary Material

Below is the link to the electronic supplementary material.

(PDF 1.93 MB)
