# Supplementary material for: Cadmium inhibitory action leads to changes in structure of ferredoxin:NADP+ oxidoreductase
Source: J Biol Phys. 2012 Feb 2;38(3):415–28. doi: 10.1007/s10867-012-9262-z (PMC3388194; doi:10.1007/s10867-012-9262-z)
Supplement: Supplementary file 1 — (PDF 1.93 MB) [file 10867_2012_9262_MOESM1_ESM.pdf]

supplementary
Click here to download attachment to manuscript: Supplementary_new.doc
Click here to view linked References

Supplementary

S.1. Circular dichroism

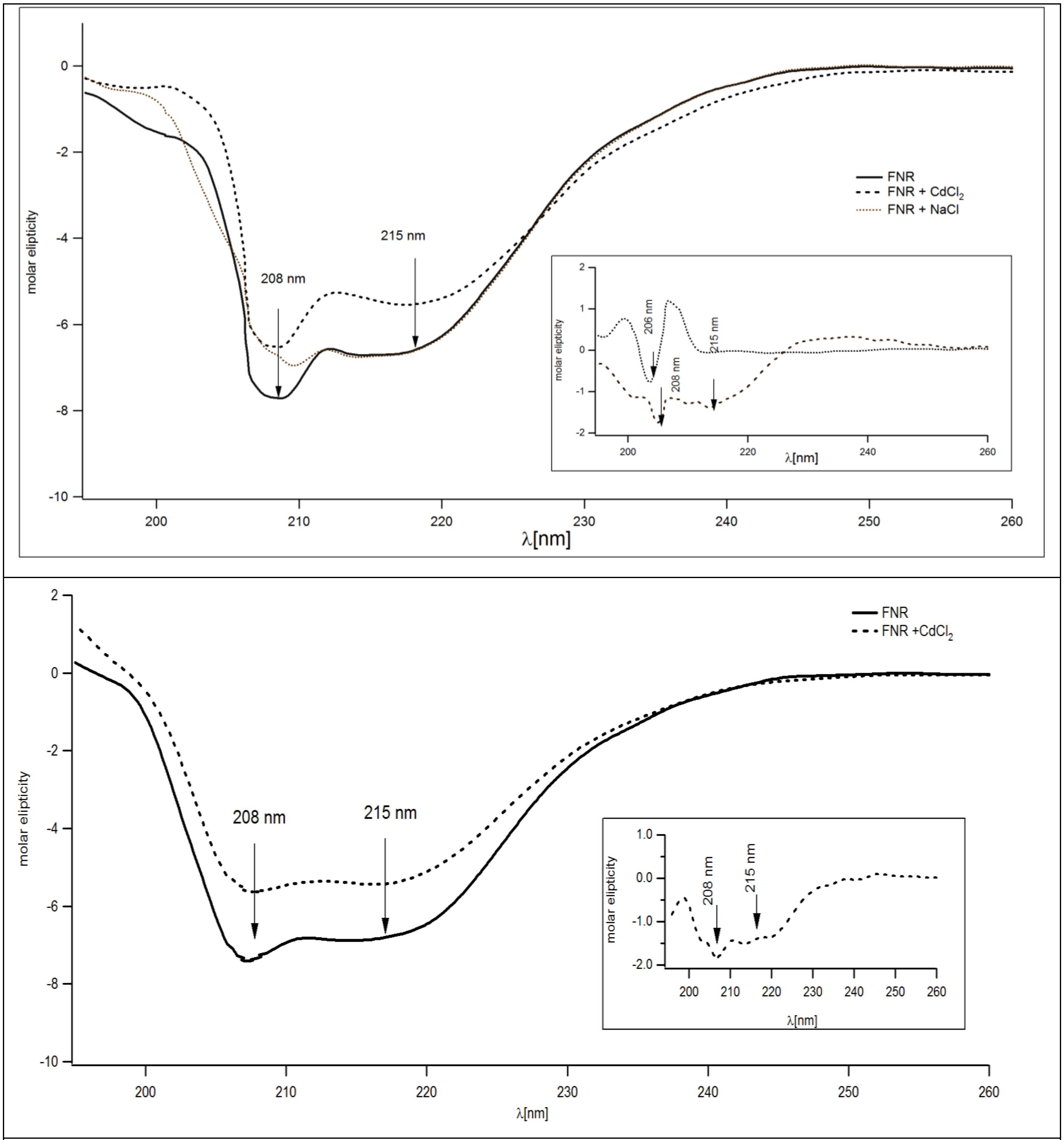

Fig.S1. Circular dichroism spectra of FNR (5.5 μM) in the presence of $CdCl_2$ at pH 8.7 and pH 7.0 A: FNR treated with 4 mM $CdCl_2$ or 8 mM NaCl in 25 mM Hepes/NaOH pH 8.7 for 15 min in room temperature. Main window – original spectra of both control and cadmium-treated FNR, insert – difference spectra constructed by substraction of the spectrum in presence of cadmium from the control spectrum. Arrows indicate the changes of the greatest magnitude. B:.FNR treated with 4 mM $CdCl_2$ in 25 mM Hepes/NaOH pH 7.0 for 15 min in room temperature. Main window - original spectra of both control and cadmium-treated FNR, insert – difference spectrum constructed by substraction of the spectrum in

presence of cadmium from the control spectrum. Arrows indicate the changes of the greatest magnitude

Circular dichroism measurement is the technique which is usually applied with success for determination of secondary structure of proteins. However, in certain conditions a good quality spectrum, allowing precise analysis, is impossible. This is in our conditions, when phosphate buffer cannot be applied (as forming unsoluble cadmium phosphate). Application of cadmium-safe buffers, as

Tris or Hepes, reduces a reliable range of measurement in the UV region up to 200-205 nm. For such range it is almost impossible to get reliable fit of secondary structure content. For accurate determination a positive dichroism peak of helix (190 nm), beta-sheet (maximum around 195 nm), but also negative dichroism peak of unstructured peptide should be measured. Without these details, fitting base on negative maxima at 209 and 222 nm (α-helix), 215 nm (β-sheet) and very week positive dichroism of unstructured chain is with very high error.

However, comparison of the spectra gives some indication if the secondary structure is impacted at all by cadmium treatment, both at pH 8.7 and 7.0 (Fig.S1). The observed change is not the effect of ionic strength increase, as confirmed by measurement with respective concentration of NaCl (Fig.S1).

S.2. Membrane-caused alteration in FNR inhibition by cadmium

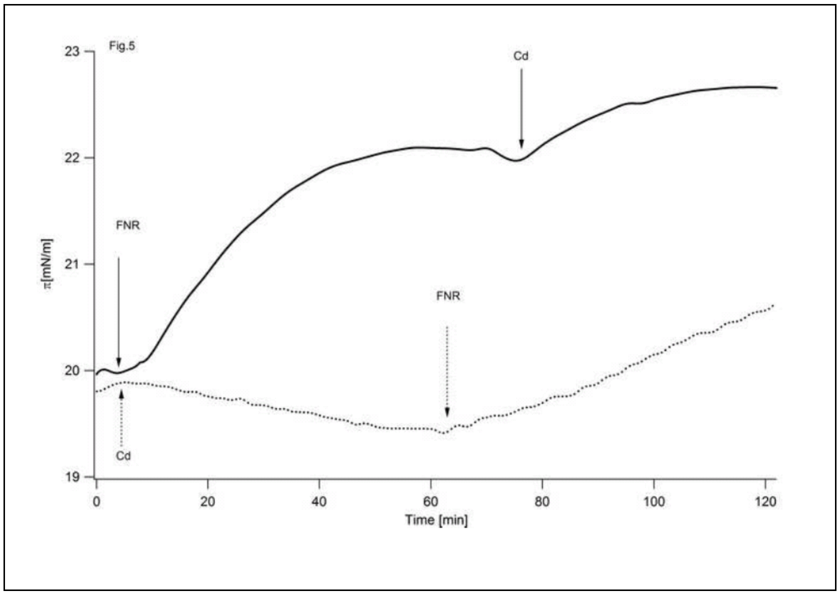

Fig.S2. Influence of $CdCl_2$ (0.2 mM) on FNR (67.25 nM) interaction with DGDG monolayer, deposited on surface of water phase, buffered with 25 mM Hepes/NaOH, pH 8.7. Arrows indicate the order of injection of FNR and cadmium solution beneath lipid monolayer.

As it has been already shown (Grzyb et al. 2008) FNR could bind to model membrane and such interaction influences significantly FNR structure. Such interaction is a probable model of one of FNR states in vivo. This is why we also attempted to check how the cadmium influence is impacted by presence of model membranes.

In this part of our research, first we checked if cadmium changed the binding of FNR to the membrane. In the experiment FNR was injected beneath monolayer (the Langmuirr-Bloggett type) of DGDG, and the significant increase in surface pressure was observed (Fig. S2, solid line, 3 min), as it has been shown already (Grzyb et al. 2008). $CdCl_2$ was introduced beneath the monolayer with FNR, after 50 min, when surface pressure stabilized. The introduced solution caused few minutes destabilization followed by 10 min increase in and stabilization. When $CdCl_2$ was injected beneath DGDG monolayer (Fig. S2, dashed line, 3 min), the surface pressure slowly decreased. Such a decrease might be an effect of interaction between cadmium and the hydrophilic lipid heads, increasing ordering. 30 min after $CdCl_2$, the FNR solution was introduced beneath that monolayer, and caused the increase in surface pressure comparable to changes observed without cadmium in the solution beneath monolayer.

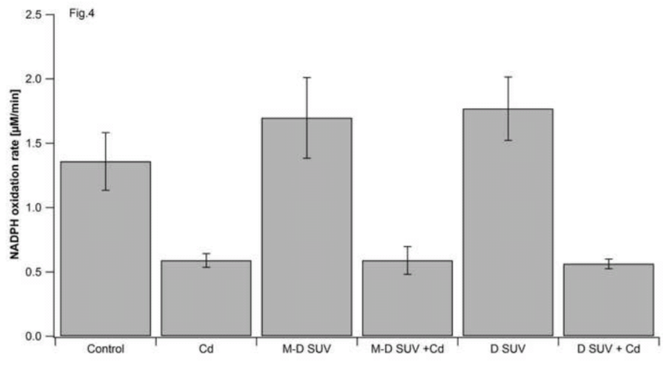

Fig.S3.Influence of CdCl2 on FNR activity, in dependence of lipids. FNR (0.2 µM) was preincubated with lipids (75 µM), cadmium (1.25 mM) or lipids and cadmium, for 30 min in 40 mM Hepes/NaOH, pH 8.7, room temperature. D-SUV – small unilamellar vesicles, 100% DGDG; M/D SUV – small unilamellar vesicles, 30%-MGDG, 70%

DGDG. Reaction was started by addition of incubation mixture to cuvette with reaction mixture; mixture contained finally 100 µM NADPH, 25 µM DBMIB, 0.02 µM FNR in 40 mM Tris/HCl pH 8.7

The results, taken together, allowed to conclude that cadmium presence increases slightly possibility of FNR attachment to membrane, most probably by changing the membrane properties – increasing amount of places available for protein to incorporate.

The incorporation of FNR into membrane cause increase of activity (as it has been also shown elsewhere (Grzyb et al. 2008) of about 25 and 29% (for MGDG-DGDG or DGDG liposomes, respectively, Fig.S3). Such activated enzyme is also more sensitive for inhibition - cadmium reduced the FNR activity to a greater extent than without liposomes. For the conditions applied, after incubation with $CdCl_2$ only, the enzyme activity was reduced by 57%, while in the presence of both $CdCl_2$ and DGDG (or MGDG-DGDG) liposomes the activity was reduced by 78% and 76%, respectively. This might suggest that liposomes did not compete with cadmium for binding sites, but stabilize the FNR structure allowing metal ions to penetrate deeper parts of the enzyme. That is of the great importance for extrapolation of our findings to *in vivo* situation, as most probably much lower concentrations of cadmium will be necessary to cause inhibitory effect.

S.4. Fluorescence

The figure S4 illustrates changes in tryptophane steady-state fluorescence, mentioned in main part of paper.

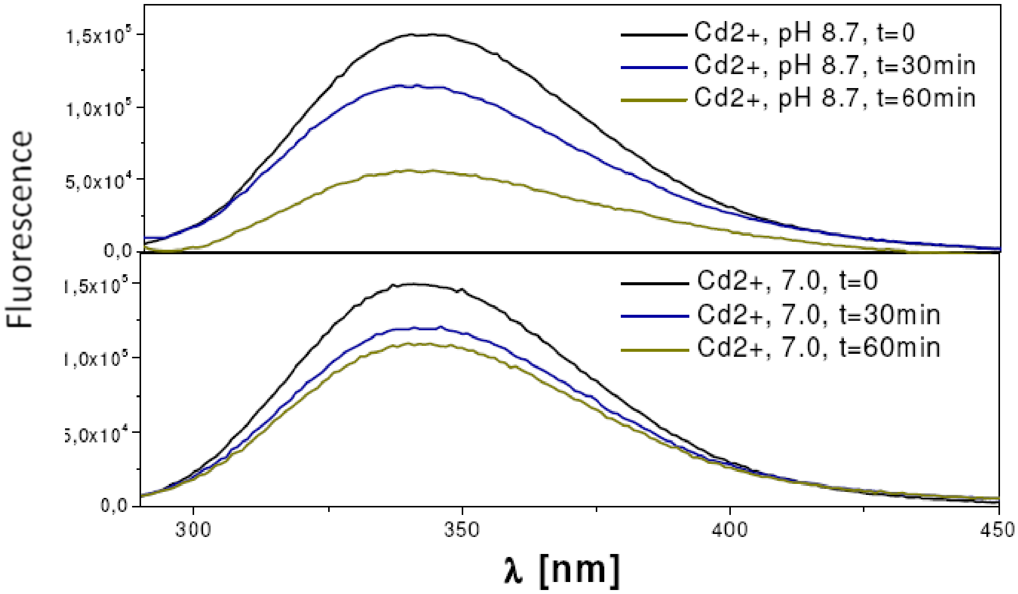

Fig.S4. Changes in the fluorescence of the Trp residues of FNR (1 μM) after cadmium (1.25 mM) treatment for 30 and 60 min in 40 mM Tris/HCl pH 8.7 or 7.0. Spectra were measured for excitation at 280 nm.

S.4. Supplementary experimentals

S.4.1. Circular dichroism

The cirular dichroism measurement is described in the main part of paper.

S.4.2. Monomolecular lipid layer on air-water interphase.

The monomolecular layers of lipids were formed from 20 μl of chloroform stock solution of lipids (DGDG or MGDG, 5 mM), as described in related experiments in Gagos et al. 2005. The water phase was buffered with 25 mM Hepes/NaOH buffer pH 7.0 or 8.7, and gently stirred with a magnetic stirrer all the time. The pH was chosen to compare the situation with inhibitory action of cadmium or the lack of such inhibition. Although greater effect of cadmium is observed in pH 8.7 than in pH 8.7, the Hepes buffering range does not cover such high pH. On the other hand, at pH 8.7 the effect of cadmium is still significant (Grzyb et al. 2010).

Since the monomolecular layer experiment is parallel to FTIR of FNR incorporated in the lipid membrane, we need a buffer which does not have absorbance in spectral region of Amide I, what is secured by the use of Hepes. The volume of the buffer in a Teflon dish was 12 ml. Surface pressure was monitored by a NIMA Technology tensiometer, model PS3 (Coventry, UK). Starting surface pressure was set ca. 20 mN/m. The FNR solution was injected by Hamilton syringe beneath the monolayer. Cadmium chloride was introduced beneath monolayer by injection of small volume of water stock solution (50 mM), when the surface pressure stabilized after injection of FNR. In second variant of experiment, cadmium chloride was injected first, and FNR was introduced beneath monolayer after stabilization of surface pressure. The changes in the surface pressure was monitored and recorded every 3 s. The whole set – the dish and the tensiometer – was closed in a container filled with argon atmosphere. Relative humidity was 100%.

S.4.3. Liposome preparation

Small unilamellar liposomes (SUV) were formed from multilamellar liposomes (MLV). For this purpose, a chloroform stock solution of DGDG, or a mixture MGDG:DGDG (30 mol% of MGDG) were placed in a glass tube, dried with a stream of nitrogen and placed under vacuum for 1 h. Then, a 25 mM Hepes buffer, pH 8.7 was

added, and the tube was vortexed for 4-5 min. The MLV were sonicated for 10 min (P=20 W, Ultrasonic homogenizer, Cole Parmer Instruments, USA) and SUV were created (manifested by the change from a white suspension to an opalising, colourless solution). The final concentration of lipids in liposome preparation was 1.4 mM.

S.5. Supplementary references

Gagoś, M., J. Gabrielska, M. Dalla Serra, Gruszecki, W.I.: Binding of antibiotic amphotericin B to lipid membranes: monomolecular layer technique and linear dichroism-FTIR studies. Mol. Membrane Biol. 22, 433-42 (2005.)

Grzyb, J., Waloszek, A., Bojko, M., Strzałka, K.: Ferredoxin:NADP+ oxidoreductase as a target of Cd2+ inhibitory action – Biochemical studies. Phytochemistry 72, 14-20 (2011)

Grzyb, J., Gagos, M., Gruszecki, W.I., Bojko, M., Strzałka, K.: The interaction of ferredoxin-NADP+ oxidoreductase with model membranes. Biochim. Biophys.Acta - Biomembranes 1778, 133-142 (2008)
